# Defining the Cholesterol Lowering Mechanism of Bergamot (*Citrus bergamia*) Extract in HepG2 and Caco-2 Cells

**DOI:** 10.3390/nu13093156

**Published:** 2021-09-10

**Authors:** Yunying Huang, Restituto Tocmo, Mirielle C. Nauman, Monica A. Haughan, Jeremy J. Johnson

**Affiliations:** 1 Department of Pharmacy Practice, College of Pharmacy, University of Illinois at Chicago, Chicago, IL 60612, USA; yyhuanggmu@outlook.com (Y.H.); rttocmo@uic.edu (R.T.); mnauma6@uic.edu (M.C.N.); mhaughan@uic.edu (M.A.H.); 2 Department of Pharmacy, The Fifth Affiliated Hospital of Guangzhou Medical University, Guangzhou 510700, China; 3 Department of Medicinal Chemistry and Molecular Pharmacology, Purdue University, West Lafayette, IN 47907, USA

**Keywords:** bergamot, neohesperidin, brutieridin, cholesterol, *Citrus bergamia*

## Abstract

Bergamot, a Mediterranean citrus fruit native to southern Italy, has been reported to have cholesterol-lowering properties; however, the mechanism of action is not well understood. Due to structural similarities with 3-hydroxy-3-methylglutaryl-coenzyme A reductase (HMGCR) inhibitors, it has been proposed that the phenolic compounds in bergamot may also inhibit HMGCR. Statins are widely used for their cholesterol-lowering properties; however, they are not universally well tolerated, suggesting there is a need to identify novel cholesterol-lowering strategies. In the present study, we investigated bergamot fruit extract (BFE) and its principal components (neoeriocitrin, naringin, neohesperidin, melitidin, and brutieridin) for their ability to regulate cholesterol levels in HepG2 and Caco-2 cells. BFE at increasing concentrations decreased the levels of total and free cholesterol in HepG2 cells. BFE and its constituents did not directly inhibit HMGCR activity. However, BFE and neohesperidin decreased HMGCR levels in HepG2 cells, suggesting that neohesperidin and BFE may downregulate HMGCR expression. An increase in AMP-kinase phosphorylation was observed in BFE and neohesperidin-treated cells. In Caco-2 cells, brutieridin exhibited a significant reduction in cholesterol uptake and decreased the level of Niemann-Pick C1 Like 1, an important cholesterol transporter. Taken together, our data suggest that the cholesterol-lowering activity of bergamot is distinct from statins. We hypothesize that BFE and its principal constituents lower cholesterol by inhibiting cholesterol synthesis and absorption.

## 1. Introduction

For decades, it has been widely recognized that lowering cholesterol reduces atherosclerotic cardiovascular disease (ASCVD) risk [1,2]. A 38.7 mg/dL (1.0 mmoL/L) reduction in low-density lipoprotein cholesterol (LDL-C) lowers the 5-year incidence of major coronary events, coronary revascularizations, and ischemic stroke by ~24% [1]. The most widely available cholesterol lowering therapies for normalizing elevated blood cholesterol are statin drugs. Statins competitively inhibit the activity of 3-hydroxy-3-methylglutaryl coenzyme A (HMG-CoA) reductase (HMGCR), the rate-limiting step in mevalonate biosynthesis, a key intermediate in cholesterol metabolism. Many patients do not attain their risk-based target LDL-C level with a statin therapy set for either a high risk (LDL-C target <100 mg/dL [2.59 mmoL/L]) or very high-risk cases (LDL-C target <70 mg/dL [1.81 mmoL/L]) [3]. For these patients, the American College of Cardiology and the American Heart Association (ACC/AHA) multisociety guideline generally recommends increasing the statin dose or initiating statin therapy in combination with another lipid-lowering agent [4]. One concern regarding statins is the safety and tolerability of high-dose statin therapy with known adverse effects that may include muscle (e.g., myalgia, myositis/myopathy, and rhabdomyolysis) and liver complications (e.g., transaminase elevation and hepatic failure). While these are rare at standard doses, the prevalence may vary between statins and increase at higher doses [5]. Due to these potential adverse effects, there are some limitations with statins, suggesting a need for alternative therapeutic approaches.

Dietary polyphenols, in particular flavonoids, exhibit a variety of significant biological functions, including protection against atherosclerosis, oxidative stress, and degenerative diseases [6]. Studies suggest that most of the biological functions of flavonoids can be attributed to their intrinsic antioxidant capabilities [7,8]. This observation provides the rationale for the optimal use of flavonoids as alternative approaches to treat hyperlipidemia and cardiovascular complications. Bergamot (*Citrus bergamia*), an endemic plant growing in Calabria (Southern Italy), has a particular composition and high content of glycosylated flavanones and flavones [9]. Among citrus plants, bergamot shows the highest content of flavonoids in juice and peels derived from its fruits [10]. Bergamot peel contains the characteristic *Citrus* species flavanone rutinosides and neohesperosides derived from naringenin, eriodictyol, and hesperetin. Neoeriocitrin, naringin, and neohesperidin are the flavanone-O-glycosides found in the highest amounts in bergamot juice (i.e., 257.0–295.8, 248.1–274.6, and 206.6–235.7 mg/L, respectively) [11]. 

The glutaryl derivatives of hesperidin and naringin (brutieridin and melitidin) contain a 3-hydroxy-3-methylglutaryl moiety with a structural similarity to the natural substrate of HMGCR and are likely to exhibit statin-like activity [12]. Preclinical and clinical studies have provided evidence that different forms of orally administered bergamot can reduce total cholesterol (TC) and LDL-C [9,13,14,15,16,17]. Although clear evidence accounts for a beneficial role of supplementation with BFE in hyperlipidemic patients, the molecular mechanism contributing to this effect still needs to be clarified. The present experiments were performed to investigate the effect of BFE on the biomarkers of cholesterol metabolism. During our evaluation we used HepG2 cells, a human hepatoma cell line, and Caco-2 cells, a human colon carcinoma cell line, which are accepted models for the study of cholesterol metabolism in the liver and intestine. HepG2 cells are highly differentiated and display many of the genotypic features of the normal liver cells [18]. Caco-2 cells display morphological and physiological similarities to mature enterocytes in the small intestine, and are suitable for the study of cholesterol absorption [19].

## 2. Materials and Methods

### 2.1. Materials 

Bergamonte^®^ *Citrus bergamia* Risso (38% BPF)-lot No. 2602-19 (BFE-A), Bergamonte^®^ Full Spectrum Bergamot Extract (*Citrus bergamia* Risso)-Lot No. 1401-19 (BFE-B), and Bergamonte^®^ Full Spectrum Bergamot Extract (*Citrus bergamia* Risso)-Lot No. 1209-18 (BFE-C) were acquired from HP Ingredients (Bradenton, FL, USA). BFEs are bergamot fruit extracts from Calabria Italy. Neoeriocitrin, naringin, and neohesperidin were acquired from Chengdu Biopurity Phytochemicals Ltd. (Chengdu, China). Dulbecco’s modified Eagle’s medium (DMEM), penicillin/streptomycin and trypsin/ethylenediaminetetraacetic acid (EDTA) were purchased from Life Technologies Co. (Grand Island, NY, USA). HMGCR activity/inhibitor screening kit was purchased from Biovision (Milpitas, CA, USA). The antibodies against HMGCR, LDL receptor, ATP-binding cassette sub-family G member 5 (ABCG5), ATP-binding cassette sub-family G member 8 (ABCG8) and β-actin were bought from ProteinTech (Rosemont, IL, USA). The antibodies against the 5′ adenosine monophosphate-activated protein kinase (AMPK), phospho-AMPK (Thr172), acetyl-coA carboxylase (ACC), and phospho-ACC (Ser79) were purchased from Sigma-Aldrich (St. Louis, MO, USA). The antibody against Niemann-Pick C1 Like 1 (*NPC1L1*) was purchased from Cell Signaling Technologies (Danvers, MA, USA). Mini-PROTEAN^®^ TGX™ pre-cast gels 4–15% were purchased from Bio-Rad (Hercules, CA, USA).

### 2.2. Isolation of Brutieridin and Melitidin 

Brutieridin and melitidin were isolated using a semi-preparative chromatography system (Pure Chromatography System, Buchi Corporation, New Haven, DE, USA). Brutieridin and melitidin in dried BFE (50 mg/mL) were separated on a 25 cm × 21.2 mm, 5 μm Discovery^®^ C18 column (Supelco, Bellefonte, PA, USA) column. The mobile phases were high performance liquid chromatography (HPLC) grade H_2_O with 0.2% formic acid (Solvent A) and methanol (Solvent B). Gradient elution was 0 min, 77% A; 30 min, 70% A; 35 min, 70% A, 40 min, 77% A with a flow rate of 14 mL/min. Peaks suspected to be brutieridin and melitidin [12] at 280 nm were pooled and dried under vacuum on a rotary evaporator (Buchi Corporation, New Haven, DE, USA), dissolved in methanol, and subjected to electrospray ionization-mass spectrometry (ESI-MS/MS, negative ion mode) via direct infusion. Mass spectra obtained were analyzed and/or compared to previous reports [7,12] to confirm the identities of compounds (>90% purity).

### 2.3. HPLC Analysis of Flavonoid Glycosides in BFE

BFE (4 mg) was dissolved in methanol to a concentration of 2 mg/mL and filtered through a 0.2 µm polyvinyl difluoride (PVDF) syringe filter. Samples were immediately injected into an HPLC UFLC system equipped with a photodiode array detector using a Phenomenex Kinetex^®^ C18 (250 mm × 4.60 mm i.d., 5 μm; Torrance, CA, USA) column. The mobile phases and elution gradient were the same as described in Section 2.2. Samples (5 μL) were injected at a flow rate of 0.5 mL/min at 30 °C column temperature. Peaks detected at 280 nm were identified by injecting commercially available standards or the isolated compounds in Section 2.2 using the same HPLC conditions, followed by matching of elution times and sample spiking. 

### 2.4. Cell Line and Cell Culture 

HepG2 (human hepatocellular carcinoma) and Caco-2 (human epithelial colorectal adenocarcinoma) cells were purchased from American Type Culture Collection. HepG2 cells were grown in DMEM supplemented with 10% fetal bovine serum, 1% penicillin/streptomycin, and 4 mM l-glutamine [20]. Caco-2 cells were grown in DMEM supplemented with 20% fetal bovine serum, 1% penicillin/streptomycin, 4 mM l-glutamine and 1% minimum essential medium nonessential amino acids (Gibco-BRL) [21]. The cells were cultured at 37 °C in a humidified atmosphere of 95% air to 5% CO_2_. 

### 2.5. Cellular Cholesterol Content Analysis 

HepG2 cells were seeded in 6-well-plates and incubated in DMEM in the absence or presence of different concentrations of samples or atorvastatin for 24 h. Then, cholesterol was measured using the Amplex^®^ Red Cholesterol Assay Kit (Invitrogen) according to the manufacturer’s instructions [22]. The Amplex^®^ Red assay was developed in a 96-well plate by the reaction of 50 μL of Amplex^®^ Red working solution (75 μL of a 300 μM of Amplex^®^ Red reagent and 2 U/mL of horseradish peroxidase, lactoperoxidase or myeloperoxidase) with 50 μL of samples. The reactions were incubated for 30 min at 37 °C, protected from light. After incubation, fluorescence was measured in a microplate reader at 530 nm and 590 nm excitation and emission wavelengths, respectively. The TC content was determined by measuring the cholesterol concentration in the presence of cholesterol esterase. To measure free cholesterol, cholesterol esterase was omitted from the assay. 

### 2.6. HMG-CoA Reductase Activity Assay

The activity of HMGCR was measured according to the instructions of the HMGCR Activity/Inhibitor Screening Kit (BioVision Inc., Milpitas, CA, USA) [23]. Briefly, HMGCR, BFE, and its principal components were added to a 96-well plate. For enzyme control, HMGCR was utilized without study agents to provide baseline activity of HMGCR. The reaction was initiated with the HMG-CoA, NADPH, and HMGCR assay buffer. Immediately, the absorbance was measured kinetically at OD 340 nm in a microplate reader.

### 2.7. Western Blotting

Cell extracts were digested in lysis buffer containing protease and phosphatase inhibitors. Total proteins were isolated and quantified by Pierce™ BCA protein assay (Thermo Fisher Scientific, Rockford, IL, USA). Western blot was performed following a previously described method with modifications [24]. The cell lysates (50 μg/lane) were mixed with 6× loading buffer and boiled for 5 min before loading. Proteins were separated by SDS-PAGE and transferred to PVDF membranes. Membranes were blocked with 5% bovine serum albumin or nonfat dry milk, then incubated with primary antibodies at 4 °C overnight. After washing three times with tris-buffered saline with 0.1% Tween 20 (TBST), membranes were incubated with appropriate secondary antibodies and bands were visualized using an enhanced chemiluminescence (ECL) kit (Pierce Biotechnology, Rockford, IL, USA) using a FluorChem E imager (ProteinSimple, San Jose, CA, USA). Band intensity was quantified using Image J software. 

### 2.8. In Vitro NBD Cholesterol Uptake 

The fluorescent analogue, 22-(N-(7 nitrobenz-2-oxa-1,3-diazol-4-yl) amino)-23,24-bisnor-5-cholen-3β-ol (NBD cholesterol), has previously been demonstrated to trace cholesterol absorption in vitro and in vivo, and therefore represents a good tool for exploring cholesterol uptake using a nonradioactive fluorescent alternative [25]. NBD cholesterol stock solution was prepared according to a previous method with modification. Caco-2 cells were incubated in Host Based Security System (HBSS) containing NBD cholesterol for 1 h at 37 °C in a humidified atmosphere of 5% CO_2_ in air. Cells were washed twice in HBSS to remove any NBD cholesterol that was not associated with the cells, and then the fluorescence was measured at excitation and emission wavelengths of λex = 485 nm and λem = 535 nm, respectively. 

### 2.9. Statistical Analysis

All the data were expressed as mean values ± standard error (SE). Differences between the groups were compared with Statistical Package for Social Science (SPSS) using one-way analysis of variance (ANOVA), and post hoc comparisons were evaluated using the Dunnett’s test. All statistical tests were 2-sided, and a significant level was set at *p* < 0.05.

## 3. Results

### 3.1. Flavonoid Glycoside Contents of BPF

**Figure 1:**
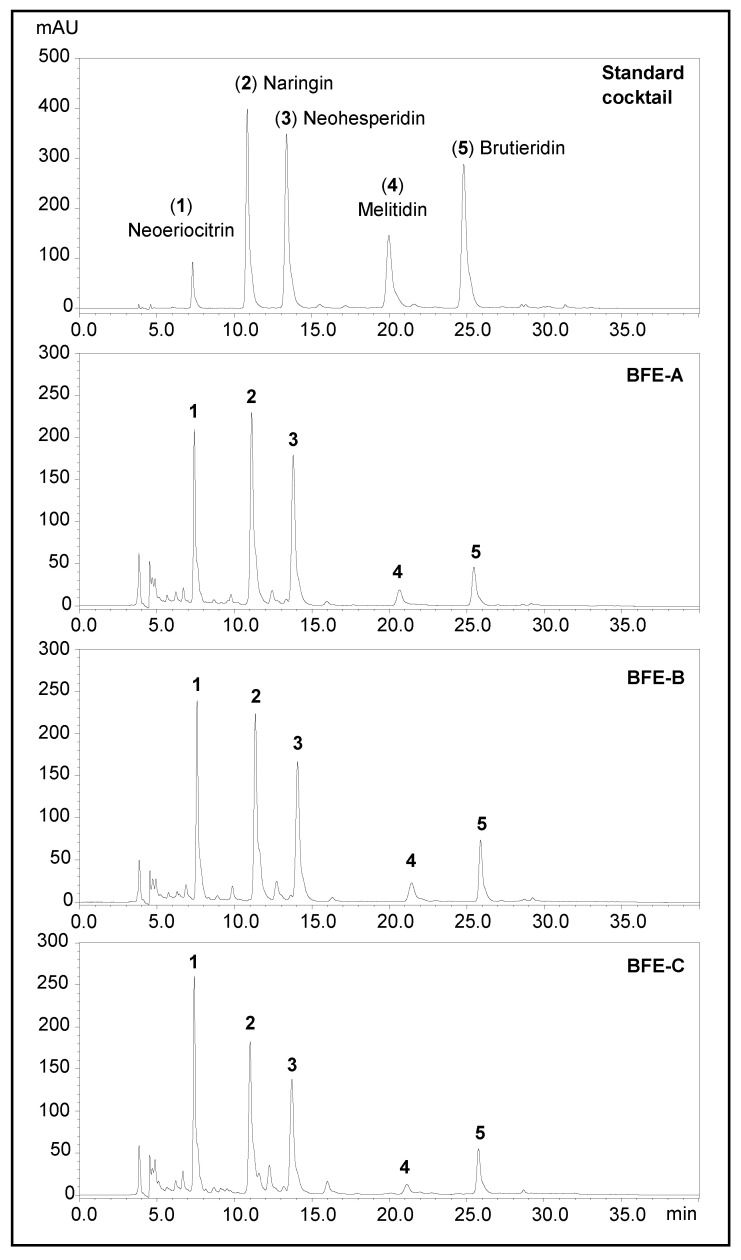
Representative HPLC chromatograms of standardized bergamot fruit extract (BFE).

BPFs A, B, and C all contained similar profiles of flavonoid glycosides (FG, Figure 1). BPF C contained significantly lower levels of naringin and melitidin, resulting in a much lower total FG (30 ± 4.8%) than BPFs A (40 ± 3.2%) and B (37 ± 5.9%) (Table 1). BPFs A and B had similar levels of neoeriocitrin (10%), naringin (12%), and neosheperidin (12.5%). Melitidin and brutieridin in BPF-A were 46% and 43% lower in BPF-A than in BPF-B. In general, our results for all three samples agreed with the values indicated in the certificate of analysis (CoA) of the commercial extracts.

### 3.2. Effects of BFE on Cholesterol Content in HepG2 Cells 

**Figure 2:**
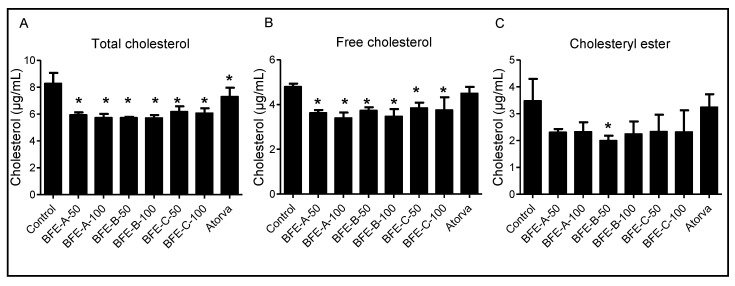
The total cholesterol, free cholesterol, and cholesterol ester in HepG2 cells treated with 50 and 100 μg/mL BFE. (**A**), total cholesterol; (**B**), free cholesterol; (**C**), cholesterol ester. Results are from four independent experiments and expressed as the mean ± SE. Significant differences between values were determined by the one-way ANOVA. *: *p* < 0.05 compared with control group. BFE-A-50, bergamot fruit extract A 50 μg/mL; BFE-A-100, bergamot fruit extract A 100 μg/mL; BFE-B-50, bergamot fruit extract B 50 μg/mL; BFE-B-100, bergamot fruit extract B 100 μg/mL; BFE-C-50, bergamot fruit extract C 50 μg/mL; BFE-C-100, bergamot fruit extract C 100 μg/mL; Atorva, atorvastatin.

The Amplex Red Cholesterol Assay quantified the total cholesterol (TC), free cholesterol (FC) and cholesterol esters (CE) and revealed that increasing concentrations of BFE in HepG2 cells decreased the levels of TC and FC compared with the control group (*p* < 0.05) (Figure 2), suggesting that BFE may be an efficient regulator of cholesterol metabolism in vitro. 

### 3.3. BFE and Its Principal Components Act Independently of HMGCR Activity 

**Figure 3:**
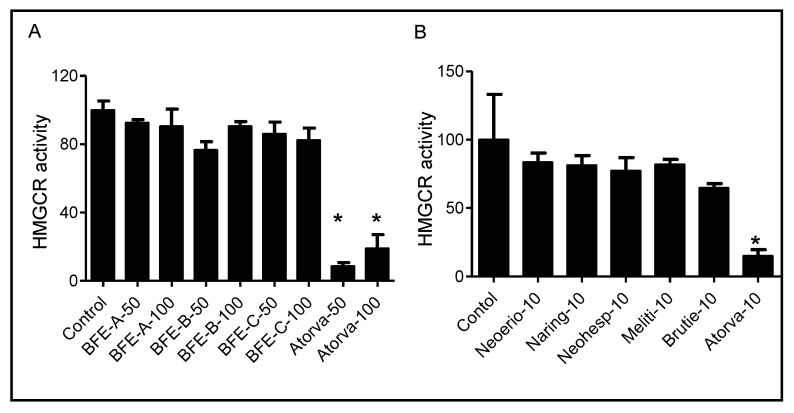
Effect of BFE (**A**) and its principal components (**B**) at indicated concentrations on HMGCR activity in vitro. Results are from three independent experiments and expressed as the mean ± SE. *: *p* < 0.05 compared with control group. BFE-A-50, bergamot fruit extract A 50 μg/mL; BFE-A-100, bergamot fruit extract A 100 μg/mL; BFE-B-50, bergamot fruit extract B 50 μg/mL; BFE-B-100, bergamot fruit extract B 100 μg/mL; BFE-C-50, bergamot fruit extract C 50 μg/mL; BFE-C-100, bergamot fruit extract C 100 μg/mL; Atorva-50, atorvastatin 50 μM; Neoerio-10, neoeriocitrin 10 μM; Naring-10, naringin; Neohesp-10, neohesperidin; Meliti-10, melitidin; Brutie-10, brutieridin; Atorva-10, atorvastatin 10 μM.

HMGCR catalyzes the conversion of HMG-CoA to mevalonate, which is the rate-limiting step in the synthesis of cholesterol and other isoprenoids [26]. To determine whether the BFE and its principal components induced antihyperlipidemic activity was dependent on HMGCR, the enzyme activity was determined using a cell-free biochemical assay. Treatment with BFE and its principal components resulted in negligible changes in HMGCR activity (*p* > 0.05) (Figure 3). These results indicated that BFE and its principal components exhibited cholesterol-lowering activity via a mechanism independent of inhibition of HMGCR. These results suggest that the BFE and its principal components display a mechanism distinct from the cholesterol-lowering statin drugs. 

### 3.4. Effect of BFE and Its Principal Components on Cholesterol Biosynthesis Related Protein Expression 

**Figure 4:**
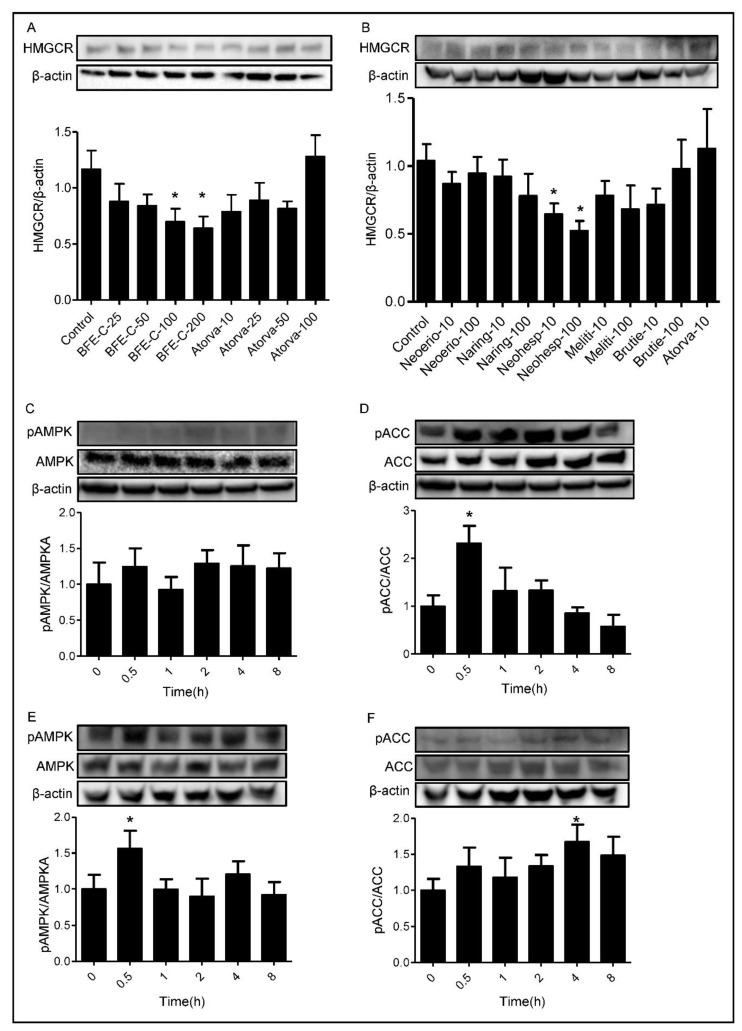
Effect of BFE and its principal components on cholesterol biosynthesis related protein expression. (**A**,**B**), HepG2 cells were treated with BFE and its principal components at the indicated concentrations for 24 h. (**C**,**D**), HepG2 cells were treated with 100 μg/mL BFE for the indicated time. (**E**,**F**), HepG2 cells were treated with 100 μM neohesperidin for the indicated time. Protein was detected by western blot analysis and quantified by densitometric analysis. BFE and neohesperidin treatments increase pAMPK/AMPK and pACC/ACC protein expression. *: *p* < 0.05, compared with control group.

We further investigated whether BFE and its principal components mediated HMGCR expression in HepG2 cells. Increasing concentrations of BFE and its principal components were used to treat HepG2 cells over a 24 h period. Figure 4 shows that 100 μg/mL.

BFE and 100 μM neohesperidin inhibited HMGCR protein expression by 40.2% and 49.6%, respectively (*p* < 0.05). This mechanism of decreased protein expression is distinct from statins that lower cholesterol through direct inhibition of HMGCR.

In the cholesterol synthesis pathway, AMPK inhibits the conversion of HMG-CoA to mevalonate by down-regulating HMGCR [27]. To investigate the effects of BFE and neohesperidin on the expression of AMPK, phospho-AMPK, ACC, and phospho-ACC, HepG2 cells were treated with BFE and neohesperidin. As shown in Figure 4C,D, phospho-AMPK expressions were time-dependently elevated by 24.6% and 56.4% in response to BFE and neohesperidin treatments for 30 min. In parallel to increased phospho-AMPK expression, phospho-ACC was increased (*p* < 0.05). These findings suggest that BFE and neohesperidin induced activation of AMPK, which may attenuate hepatic lipid accumulation. 

### 3.5. Effect of BFE Principal Components on Protein Expression of LDL Receptor

**Figure 5:**
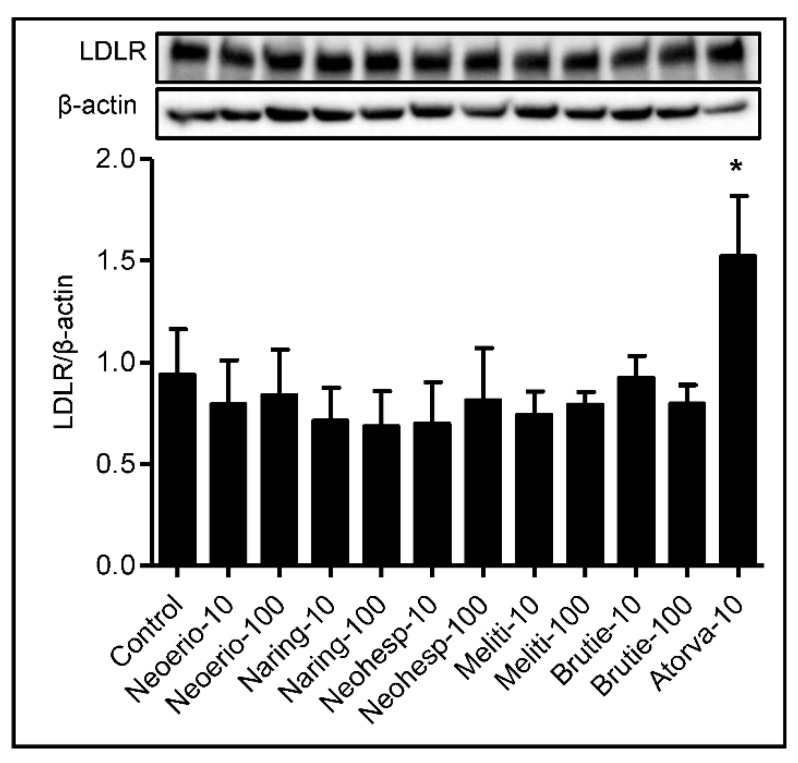
Effect of BFE principal components on protein expression of the LDL receptor. HepG2 cells were treated with indicated concentrations of BFE principal components for 24 h. After treating cells, we performed Western blot for the LDL receptor and β-actin. The intensity of the LDL receptor band was densitometrically measured and normalized to the protein expression level of β-actin. Results are from five independent experiments and expressed as the mean values ± SE. *: *p* < 0.05 compared with control group.

Uptake of cholesterol mediated by the LDL receptor plays a crucial role in lipoprotein metabolism [28]. The effect of BFE principal components on the protein expression of LDL receptor was determined, as shown in Figure 5. Treatment with BFE principal components produced no significant differences in the protein expression of LDL receptor compared to the control.

### 3.6. Effect of BFE Principal Components on Cholesterol Uptake in Caco-2 Cells 

**Figure 6:**
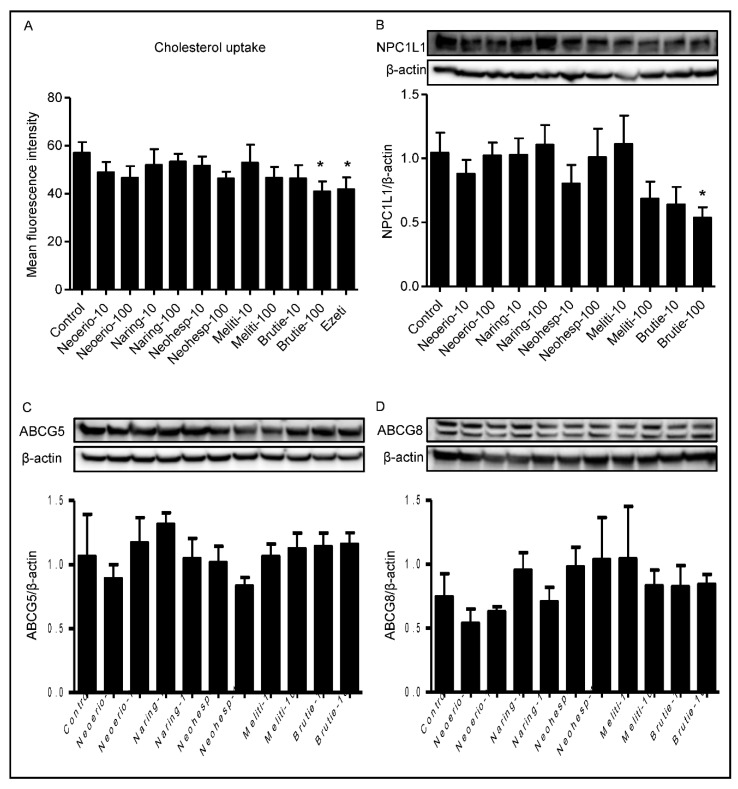
Effect of BFE principal components on cholesterol uptake (**A**) and cholesterol transport protein expression in Caco-2 cells. Cells were treated with BFE principal components (10 and 100 μM) for 24 h. (**B**–**D**), *NPC1L1*, ABCG5 and ABCG8 protein expression was detected by western blot analysis and quantified by densitometric analysis. The results are presented as mean ± SE.*: *p* < 0.05 compared with control group.

Another mechanism of cholesterol-lowering drugs includes the blocking of cholesterol absorption in the gastrointestinal tract. To evaluate this, we utilized Caco-2 cells to quantify cholesterol absorption following exposure to constituents in BFE. Interestingly, cholesterol absorption in Caco-2 cells was significantly suppressed by brutieridin compared with the control group (Figure 6). The reduced cholesterol uptake was observed at the concentration of 100 μM brutieridin (*p* < 0.05). Brutieridin inhibited cholesterol absorption in a concentration-dependent manner. 

### 3.7. Effect of BFE Principal Components on NPC1L1 Protein Expression

The *NPC1L1* protein plays a critical role in the absorption of intestinal cholesterol. Brutieridin at 100 μM induced a nearly 50% reduction of *NPC1L1* protein expression after 24 h incubation (*p* < 0.05) (Figure 6). Treatment with BFE principal components produced no significant differences in the protein expression of ABCG5 or ABCG8 compared to the control.

## 4. Discussion

Hypercholesterolemia is an important risk factor for the development of ASCVD. Consequently, inhibition of cellular cholesterol synthesis and transport, whether through medication or diet therapy, has been a primary strategy to reduce the risk of ASCVD for several years. The primary finding of this study revealed that cholesterol metabolism and processing in both the liver and intestine were affected by BFE and its principal components. These results suggest that these dietary compounds can act as signaling molecules and thus impart a cholesterol-lowering effect at both major sites of the enterohepatic circulation.

The hypolipidemic effects of bergamot have been documented for more than 10 years [14]. Most of the previous studies were performed in animal models followed by analysis of the cholesterol levels in the plasma and liver. The most consistent finding from the previous studies with bergamot is decreased plasma levels of cholesterol. The mechanisms underlying both blood cholesterol and lipids/fats lowering effects of bergamot remain to be fully understood; however, evidence suggests that the mechanism is distinct from statin drugs, contrary to previous suggestions that they share the same mechanism as statins. It is well known that statins decrease cholesterol concentrations by directly inhibiting HMGCR [29]. Importantly, BFE contains brutieridin and melitidin, which are 3-hydroxy-3-methylglutaryl derivatives of hesperetin and naringenin, respectively [12,30]. However, there is not any in vitro or in vivo studies that support this mechanism. There have been suggestions that since these compounds share a structural similarity with the natural substrate of HMGCR they possess statin-like properties by selective inhibition of HMGCR [31]. We determined that there is not a direct inhibition of HMGCR activity by BFE and its principal components (Figure 3). Although, BFE and its principal components had no effect on HMGCR activity, a decrease in total and free cholesterol was observed, which is consistent with the clinical reports on the cholesterol lowering effects of bergamot (Figure 2). This implies that the hypolipidemic activities of BFE are due to an unidentified mechanism different than those of statins.

To better understand possible targets and the cholesterol-lowering mechanism of BFE, we evaluated the expression of HMGCR. BFE and neohesperidin significantly downregulated the expression of HMGCR in HepG2 cells. HMGCR is regulated by cellular metabolic state which is thought to help the cell optimize ATP expenditure during metabolic stress. It is worth noting that AMPK serves as a key energy sensor that plays an important role in sustaining cellular energy levels and has emerged as a potential therapeutic target for metabolic diseases [32]. AMPK is an upstream kinase for critical metabolic enzymes, including HMGCR and ACC, and is activated by phosphorylation of a threonine residue within the activation segment of the α subunit (Thr^172^ in rat AMPK) [33]. AMPK blocks the conversion of HMG-CoA to mevalonate by down-regulating HMGCR [27]. In the fatty acid biosynthesis pathway, inactivation of ACC by AMPK reduces malonyl-CoA concentration, leading to stimulation of fatty acid oxidation concomitant with an increase in ß-oxidation. We treated HepG2 cells with BFE and its principal components to investigate whether they affect AMPK phosphorylation. As a result, AMPK phosphorylation was increased in HepG2 cells treated with BFE and neohesperidin, compared with those in control cells. It has been reported that phosphorylation of AMPK is involved in the attenuation the progression of adipogenesis by BFE in mesenchymal stem cells from human adipose tissue [34]. Similarly, a previous in vivo study showed that treatment with neohesperidin reduces plasma TC concentration by increasing AMPK activity in KK-Ay mice [35]. Our results suggest that BFE and neohesperidin may inhibit accumulation of cholesterol by stimulating AMPK activity in HepG2 cells. Besides biosynthesis, cholesterol uptake from the blood has key roles in maintaining cholesterol homeostasis. Uptake of cholesterol mediated by the LDL receptor plays a crucial role in lipoprotein metabolism [28]. However, we did not observe any significant effect of BFE principal components on the protein expression of LDL receptors.

Circulating plasma cholesterol concentrations depend on the integrated balance of cholesterol synthesized by the liver and on the cholesterol absorbed by the intestine [36]. In fact, cholesterol absorption rate is directly related to plasma cholesterol concentrations. Since intestinal cholesterol absorption affects the levels of cholesterol in blood circulation, the effect of BFE principal components on cholesterol uptake was determined in Caco-2 cells. Our results demonstrated that brutieridin inhibited cholesterol uptake in Caco-2 cells. Several studies suggest that intestinal cholesterol absorption is the result of cooperation among several membrane transporters, including *NPC1L1*, ABCG5, and ABCG8 [37]. However, we did not observe any significant effect of BFE principal components on the protein expression of ABCG5 or ABCG8. The present work demonstrated a novel effect of the component in the intestine, i.e., inhibition of *NPC1L1*. Our results suggest that brutieridin may decrease the cholesterol levels by inhibiting uptake via the *NPC1L1* transporter. There are seven polyphenols that have been reported to affect the activity of *NPC1L1*, through inhibition of transporter activity or expression [38]. Luteolin has been shown to inhibit *NPC1L1* expression by decreasing *NPC1L1* mRNA levels in Caco-2 cells and mouse intestinal mucosa [39]. This suggests the possibility that polyphenols exert multiple actions on *NPC1L1* activity. In recent studies of cholesterol absorption, Caco-2 cell cultures have been used as a model system [19]. Caco-2 cells are suitable for the study of cholesterol absorption as they can absorb cholesterol and share morphological and physical similarities to mature enterocytes in the small intestine. However, enterocytes are only one of the four main cell types found in the intestine, indicating that it does not entirely reflect cholesterol handling in vivo [40]. Further analysis of an in vivo study will provide a more thorough understanding of how cholesterol absorption can be modified. 

## 5. Conclusions

In conclusion, BFE and its principal constituents reduce cholesterol in a mechanism that is distinct from direct inhibition of HMG-CoA reductase, as has been suggested previous laboratory and clinical studies. This is significant as there is now evidence that bergamot fruit extract does not share the same pharmacological mechanism as the cholesterol-lowering drugs known as statins. Our results provide new insights into the molecular mechanism of the cholesterol-lowering effects of BFE. The cholesterol-lowering effect of BFE appears to be mediated through several mechanisms, including cholesterol biosynthesis in HepG2 cells and cholesterol cellular transport in Caco-2 cells. Overall, the data support the hypothesis that BFE and its principal constituents can alter cholesterol synthesis and uptake. Further studies will be focus on further evaluating these findings in animal studies. In addition, this will provide an opportunity to characterize the possible effect of the colonic metabolites of BFE for their cholesterol-lowering actions on cholesterol metabolism. BFE may represent a potential alternative therapeutic approach for lowering cholesterol, especially in subjects suffering from statin intolerance. These findings shed light on the use of BFE in reduction of overall cardiovascular disease risk.

## Figures and Tables

**Table 1:** Concentrations of flavonoid glycosides in bergamot extracts.

	A		B		C	
	Concentration (%) *	CoA (%)	Concentration (%)	CoA (%)	Concentration (%)	CoA (%)
Neoeriocitrin	10.14	9.2	10.17	9.52	10.29	ns
SD	1.20		0.36		0.29	
Naringin	12.43	12.7	11.88	12.93	8.86	ns
SD	0.47		1.07		1.06	
Neohesperidin	12.66	12.4	12.84	12.34	10.28	ns
SD	1.40		2.06		1.62	
Melitidin	1.38	1.3	2.44	2.2	0.59	ns
SD	0.42		0.19		0.10	
Brutieridin	3.27	2.7	4.74	4.5	3.95	ns
SD	0.17		0.21		0.50	
Total FG	39.89	38.3	37.34	41.48	30.35	38
SD	3.20	-	5.93	2.0	4.77	-

* *n* = 5; ns = not specified; CoA = Certificate of Analysis; SD = standard deviation.

## Data Availability

The data presented in this study are available on request from the corresponding author.
